# Temporal Trends and Sex Differences in Disability-Adjusted Life Years (DALYs), Years Lived With Disability (YLDs), and Years of Life Lost (YLLs) Attributable to Infectious Diseases in Afghanistan: Global Burden of Disease 2023 Estimates, 1999–2023

**DOI:** 10.7759/cureus.115646

**Published:** 2026-09-02

**Authors:** Ahmad Khan, Melanie Tidman

**Affiliations:** 1 Health Sciences, Andrew Taylor (A.T.) Still University, Albuquerque, USA

**Keywords:** afghanistan, dalys, infectious diseases, lower-income countries, ylds, ylls

## Abstract

This study examined 25 years (1999-2023) of Global Burden of Disease (GBD) 2023 data to understand how infectious disease burden has changed over time in Afghanistan and whether men and women are affected differently. The study analyzed disability‑adjusted life years (DALYs), years lived with disability (YLDs), and years of life lost (YLLs) for 46 infectious etiologies, generating 2,300 disease-year-sex data points for DALYs and YLDs, and 2,292 for YLLs. Annual Wilcoxon signed-rank tests showed that men had higher DALY and YLL rates for many etiologies in most years, with particularly consistent male excess from 2016 onward, whereas women had higher YLD rates for many etiologies throughout the study period. When pooled across all 25 years, the overall Mann-Whitney U test found no significant sex difference in DALYs (p=0.158) or YLLs (p=0.161), but did find a significant female excess in YLDs (p=0.017). Generalized estimating equation models (GEE), which accounted for repeated annual observations within each etiology-by-sex series, showed significant declines in burden rates over time. DALY rates declined by 5.354 units per year (95% confidence interval (CI): -8.178 to -2.530), YLL rates declined by 5.301 units per year (95% CI: -8.105 to -2.498), and YLD rates declined by 0.043 units per year (95% CI: -0.066 to -0.020; all p<0.001). Sex-by-year interactions were not statistically significant for DALYs (p=0.938), YLLs (p=0.936), or YLDs (p=0.792).

Severity-stratified heatmap analysis classified five etiologies (10.9%) as high burden, 32 etiologies (69.6%) as intermediate burden, and nine etiologies (19.6%) as low burden according to their cross-etiology mean DALY rates. *Streptococcus pneumoniae*, rotavirus, and *Klebsiella pneumoniae* were among the etiologies with the highest burden throughout the study period.

These findings suggest that Afghanistan’s infectious disease burden improved modestly over the 25-year study period. However, the improvements were unevenly distributed across etiologies. Annual comparisons identified sex differences in many years; however, GEE models found no evidence that male and female burden rates followed different linear trends over time. Targeted, sex-responsive, and etiology-focused public health strategies are still needed to decrease the burden of infectious disease in vulnerable populations.

## Introduction

Years of ongoing conflict, a compromised healthcare system with limited preventive and medical care access, and mass displacement have left Afghanistan continuing to experience a high burden of infectious diseases [1,2]. These challenges are exacerbated by limited access to basic preventive and medical care [2,3]. Even with the long-term expansion of targeted investments in immunization, maternal-child health, and primary care, infections remain a primary cause of early death and disability in vulnerable and resource‑limited populations [4-6]. Measuring the impact of infectious diseases is vital for allocating healthcare resources, setting plan priorities, and tracking progress toward universal healthcare.

The Global Burden of Disease (GBD) Study provides a standardized and systematic platform for measuring the prevalence, mortality, years of life lost (YLLs), years lived with disability (YLDs), and years lived with disability (DALYs) for a broad spectrum of diseases and injuries across different settings and time frames [7,8]. In this framework, DALYs are calculated by summing together YLLs and YLDs, capturing the combined fatal and non‑fatal mortality from specific causes [9].

Men and women differ in their risk and outcomes for infectious diseases due to behavioral and social determinants [10,11]. Assessing how infectious diseases affect men and women differently over time highlights inequities in care. This assessment will help experts develop more effective health guidelines for women, even when access to care is very limited. The GBD 2023 has data at global and national levels for various diseases and injuries, including infectious disease estimates and age‑standardized rates by calendar year, presented separately for men and women [12]. Research in Afghanistan that quantifies temporal trends and sex‑specific differences in DALYs, YLDs, and YLLs for various infectious diseases is limited. This study aims to examine infectious disease rates in Afghanistan and, if any, identify sex differences in the general population using GBD 2023 data. The results can provide practical evidence to guide Afghan health programs and their international partners in tackling sex differences in infectious disease rates.

Study objective

This study aimed to investigate temporal trends and sex differences in age-standardized DALY, YLD, and YLL rates attributable to 46 infectious disease etiologies in Afghanistan between 1999 and 2023, using GBD 2023 estimates. This study characterized overall changes in burden rates over time, described male and female differences across etiologies within each year, and evaluated whether adjusted linear temporal trends differed by sex while accounting for repeated annual observations within etiology-by-sex series.

## Materials and methods

This study is a secondary cross-sectional time-series analysis of publicly available aggregated data from the GBD 2023 Study [13]. The GBD 2023 employs a comprehensive modeling framework that uses different data sources such as systematic reviews, population-based surveys, vital registration systems, disease registries, and established modeling approaches for non-fatal outcomes and mortality for 375 diseases and injuries across 204 countries and territories [13]. For Afghanistan, age-standardized rates per 100,000 population for DALYs, YLDs, and YLLs were extracted for 46 infectious etiologies for the years 1999-2023, stratified by sex (men and women), using the GBD Results Tool on April 1, 2026. The GBD 2023 Study reports 95% uncertainty intervals; however, this analysis used only the reported age-standardized rate point estimates. Uncertainty from the underlying GBD estimation process was not propagated through the statistical analyses.

Analysis, outcome measures and variables

Three primary outcomes were examined as age-standardized rates per 100,000 population for DALYs, YLDs, and YLLs for each of the 46 infectious etiologies as defined in the GBD 2023 cause list. DALYs followed the standard GBD definition as the aggregate of YLLs (premature mortality) and YLDs (non-fatal morbidity), in accordance with standard GBD methodology. The main independent variables were calendar year, treated as a continuous variable (1999-2023), and sex (male, female) as reported in the GBD outputs. Data for Afghanistan were downloaded from the GBD Results Tool by selecting measure (DALYs, YLDs, YLLs), metric (rate), cause (infectious etiologies), location (Afghanistan), age group (age-standardized), sex (male, female), and year (1999-2023), and then exported in a format compatible with IBM SPSS Statistics, Version 31 (IBM Corp., Armonk, NY). Each observation represented a specific cause-year-sex combination (46 causes×25 years×2 sexes), which generated 2,300 disease-year-sex data points for DALYs and YLDs and 2,292 data points for YLLs, as female YLLs data for eight enteric etiology were not available in the GBD 2023 dataset in 2022.

Data processing and reproducibility

Separate GBD Results Tool downloads were obtained for DALYs, YLDs, and YLLs using the following settings: location, Afghanistan; metric, rate per 100,000 population; age group, age-standardized; sex, male and female; years, 1999-2023; and the 46 infectious etiologies included in the GBD 2023 cause hierarchy. The downloaded datasets were imported into IBM SPSS Statistics version 31 and checked for duplicate cause-sex-year combinations. For each measure, records were organized so that each row represented one cause-year-sex observation. Calendar year was coded as a continuous variable, sex was coded as male or female, and an etiology-by-sex identifier was created by combining the cause and sex variables to define the repeated series for generalized estimating equation (GEE) model analysis. No values were transformed or imputed. For YLLs, female estimates for eight enteric pathogen etiologies were not available in 2022. These observations were treated as missing and excluded only from analyses requiring those values, including the 2022 paired male-female comparison. The final analysis datasets contained 2,300 observations each for DALYs and YLDs and 2,292 observations for YLLs. The 46 infectious etiologies analyzed were: *Streptococcus pneumoniae*, Rotavirus, *Klebsiella pneumoniae*, Enteropathogenic *Escherichia coli*, Adenovirus, *Pseudomonas aeruginosa*, Enterotoxigenic *E. coli*, Influenza, Shigella, Campylobacter, other *Mycobacterium* species (non-tuberculous, non-leprosy), *Cryptosporidium*, respiratory syncytial virus, *E. coli*, *Neisseria meningitidis*, *Haemophilus influenzae*, Norovirus, *Staphylococcus aureus*, other bacterial and viral pathogens, Group B Streptococcus, *Acinetobacter baumannii*, Cholera, Mycoplasma, Chlamydia, non-polio enteroviruses, non-typhoidal *Salmonella*, other gram-negative bacteria, other fungi, Group A Streptococcus, other viral etiologies of meningitis, *Aeromonas*, *Candida* species, coagulase-negative staphylococci, *Aspergillus* species, *Entamoeba*, other *Streptococcus* species, *Listeria* monocytogenes, *Enterobacter* species, other *Acinetobacter* species, other *Klebsiella* species, *Serratia* species, *Morganella* species, *Proteus* species, *Citrobacter* species, *Clostridium difficile*, and *Legionella* species.

The non-parametric Mann-Whitney U (Wilcoxon rank-sum) test was used to compare overall DALYs, YLDs, and YLLs between men and women across all infectious etiologies, examining sex differences for each of the 46 infectious etiologies over the study period. Paired Wilcoxon signed-rank tests were conducted to compare male and female age-standardized rates for corresponding years (n=46 diseases per year, except for YLLs in 2022 where n=38 due to missing female values for eight enteric pathogens), and the resulting negative ranks (female<male), positive ranks (female>male), and ties were tabulated. For Mann-Whitney U tests, Z statistics and two-tailed p-values were reported. For Wilcoxon signed-ranks tests, the Wilcoxon W statistic and two-tailed p-values were reported. All statistical tests were two-sided, and p-values <0.05 were considered statistically significant.

Temporal trends were analyzed separately for age-standardized DALY, YLD, and YLL rates using GEE. Each model included calendar year as a continuous variable and sex and etiology as categorical predictors, with a calendar year-sex interaction to assess sex-specific differences in trends from 1999 to 2023. Repeated annual observations clustered within etiology-by-sex series were addressed using an AR(1) working correlation structure, and robust standard errors were used. Regression coefficients, 95% confidence intervals, and two-sided p-values were reported. A negative year coefficient indicated a decrease in the age-standardized rate over time. The GEE models were fitted separately for DALYs, YLDs, and YLLs with age-standardized rate as the dependent variable; calendar year, sex, etiology, and the calendar year-by-sex interaction as predictors; etiology-by-sex as the repeated-subject variable; calendar year as the within-subject variable; an AR(1) working correlation structure; and robust standard errors.

The temporal heatmap was created to visualize the distribution of etiology-specific DALY rates across all 46 infectious etiologies. The mean DALY rate for each etiology was calculated by averaging for both genders the age-standardized rates across all 25 years and was not used for inferential testing. Etiologies were then classified into three severity bands based on the cross-etiology distribution: etiologies with a mean DALY rate at or above the cross-etiology mean plus 0.5 standard deviation (SD) were classified as high burden (n=5); those at or below the mean minus 0.5 SD were classified as low burden (n=9); and all remaining etiologies were classified as intermediate burden (n=32). The overall cross-etiology mean DALY rate was 119 per 100,000 population (SD=196), yielding severity thresholds of ≥217 for high-burden and ≤21 for low-burden. Within the heatmap, a separate class-specific color gradient was applied to each severity band (red for high, blue for intermediate, and green for low-burden). Color intensity scaling was applied to within-class minimum and maximum mean DALYs rates to enhance contrast within each group. Etiologies were ordered from highest to lowest mean DALYs burden within each severity band. The heatmap was generated using Python (Matplotlib, version 3.x; Python Software Foundation, Fredericksburg, VA).

## Results

The Wilcoxon signed-ranks test was used to compare age-standardized infectious disease burden rates between women and men across 46 infectious diseases per year in Afghanistan from 1999 to 2023. Since the data did not follow a normal distribution, the evaluation was performed via a non-parametric test to assess the disease-level paired comparisons between women and men for each calendar year independently. The test evaluates whether one sex consistently has significantly higher or lower disease burdens across all 46 infectious diseases.

For DALYs - the composite measure of total disease burden - men demonstrated a higher burden than women (more lower ranks) in the majority of years throughout the study period. Statistically significant sex-based differences were observed in 2001 (W=303.000, p=0.009), 2002 (p=0.022), 2011 (p=0.001), and male DALY rates were significantly higher across infectious etiologies in most study years, including 1999-2011 and 2015-2023; the only years without statistically significant cross-etiology sex differences were 2012-2014 (all p>0.05).

Female YLD rates were significantly higher than male YLD rates across infectious etiologies in every study year from 1999 to 2023 (all p<0.001). This consistent annual pattern indicates a higher cross-etiology non-fatal infectious disease burden among females. However, it should not be interpreted as evidence of different adjusted longitudinal trends by sex because the GEE sex-by-year interaction was not statistically significant (see Table 1).

**Table 1:** Annual Sex-Based Differences in Age-Standardized Infectious Disease Burden in Afghanistan, 1999–2023 DALYs=disability-adjusted life years; YLDs=years lived with disability; YLLs=years of life lost; F=females; M=males; Neg=negative ranks, representing diseases in which the female age-standardized rate was lower than the male rate (F<M), indicating a higher male burden; Pos=positive ranks, representing diseases in which the female age-standardized rate was higher than the male rate (F>M), indicating a higher female burden; Ties=diseases with equal rates in females and males; W=Wilcoxon signed-ranks test statistic; p=asymptotic two-tailed p-value. Negative ranks, positive ranks, and ties are presented as number (percentage) of matched infectious diseases. For example, 34 (73.9%) indicates that 34 of 46 matched infectious diseases had lower rates in women than in men. Each annual analysis included 46 matched infectious diseases (100.0%) for DALYs and YLDs and for YLLs in all years except 2022. The YLL analysis in 2022 included 38 matched infectious diseases (82.6% of the expected 46) because eight enteric pathogens - *Campylobacter*, Cholera, *Cryptosporidium*, *Entamoeba*, Enteropathogenic *Escherichia coli*, Enterotoxigenic *E. coli*, non-typhoidal *Salmonella*, and *Shigella* - were absent from the female GBD 2023 source data for that year. Statistical significance was defined as p<0.05; p-values <0.001 are reported as p<0.001. Data source: Global Burden of Disease (GBD) 2023, Afghanistan [13].

Year	DALYs Neg number	DALYs Neg %	DALYs Pos number	DALYs Pos %	DALYs Ties number	DALYs Ties %	DALYs W	DALYs p-value	YLDs Neg number	YLDs Neg %	YLDs Pos number	YLDs Pos %	YLDs Ties number	YLDs Ties %	YLDs W	YLDs p-value	YLLs Neg number	YLLs Neg %	YLLs Pos number	YLLs Pos %	YLLs Ties number	YLLs Ties %	YLLs W	YLLs p-value
1999	34	73.9%	12	26.1%	0	0.0%	294	0.006	0	0.0%	43	93.5%	3	6.5%	0	<0.001	34	73.9%	12	26.1%	0	0.0%	288	0.005
2000	34	73.9%	12	26.1%	0	0.0%	317	0.014	0	0.0%	43	93.5%	3	6.5%	0	<0.001	34	73.9%	12	26.1%	0	0.0%	308	0.010
2001	34	73.9%	12	26.1%	0	0.0%	303	0.009	0	0.0%	43	93.5%	3	6.5%	0	<0.001	34	73.9%	12	26.1%	0	0.0%	303	0.009
2002	34	73.9%	12	26.1%	0	0.0%	332	0.022	0	0.0%	43	93.5%	3	6.5%	0	<0.001	34	73.9%	12	26.1%	0	0.0%	329	0.020
2003	34	73.9%	12	26.1%	0	0.0%	312	0.012	0	0.0%	43	93.5%	3	6.5%	0	<0.001	34	73.9%	12	26.1%	0	0.0%	307	0.010
2004	34	73.9%	11	23.9%	1	2.2%	250	0.003	0	0.0%	43	93.5%	3	6.5%	0	<0.001	35	76.1%	11	23.9%	0	0.0%	257	0.002
2005	36	78.3%	9	19.6%	1	2.2%	179	<0.001	1	2.2%	42	91.3%	3	6.5%	33	<0.001	36	78.3%	9	19.6%	1	2.2%	176	<0.001
2006	38	82.6%	6	13.0%	2	4.3%	114	<0.001	1	2.2%	42	91.3%	3	6.5%	34	<0.001	39	84.8%	6	13.0%	1	2.2%	118	<0.001
2007	42	91.3%	4	8.7%	0	0.0%	70	<0.001	1	2.2%	41	89.1%	4	8.7%	37	<0.001	42	91.3%	4	8.7%	0	0.0%	69	<0.001
2008	42	91.3%	3	6.5%	1	2.2%	55	<0.001	1	2.2%	39	84.8%	6	13.0%	35	<0.001	42	91.3%	4	8.7%	0	0.0%	57.5	<0.001
2009	43	93.5%	2	4.3%	1	2.2%	51	<0.001	1	2.2%	40	87.0%	5	10.9%	35	<0.001	43	93.5%	2	4.3%	1	2.2%	49	<0.001
2010	37	80.4%	7	15.2%	2	4.3%	124	<0.001	1	2.2%	39	84.8%	6	13.0%	32.5	<0.001	38	82.6%	7	15.2%	1	2.2%	120	<0.001
2011	32	69.6%	12	26.1%	2	4.3%	214.5	0.001	2	4.3%	38	82.6%	6	13.0%	42.5	<0.001	32	69.6%	12	26.1%	2	4.3%	199	<0.001
2012	26	56.5%	18	39.1%	2	4.3%	367.5	0.137	2	4.3%	36	78.3%	8	17.4%	37.5	<0.001	26	56.5%	18	39.1%	2	4.3%	349	0.088
2013	22	47.8%	23	50.0%	1	2.2%	462	0.531	2	4.3%	37	80.4%	7	15.2%	25.5	<0.001	24	52.2%	21	45.7%	1	2.2%	448.5	0.436
2014	25	54.3%	20	43.5%	1	2.2%	430	0.323	2	4.3%	40	87.0%	4	8.7%	31	<0.001	25	54.3%	20	43.5%	1	2.2%	414	0.243
2015	26	56.5%	19	41.3%	1	2.2%	310.5	0.019	2	4.3%	39	84.8%	5	10.9%	29.5	<0.001	26	56.5%	19	41.3%	1	2.2%	295	0.012
2016	37	80.4%	6	13.0%	3	6.5%	82	<0.001	2	4.3%	37	80.4%	7	15.2%	39	<0.001	40	87.0%	5	10.9%	1	2.2%	70.5	<0.001
2017	43	93.5%	3	6.5%	0	0.0%	29	<0.001	2	4.3%	39	84.8%	5	10.9%	43	<0.001	43	93.5%	3	6.5%	0	0.0%	28	<0.001
2018	45	97.8%	1	2.2%	0	0.0%	12	<0.001	2	4.3%	40	87.0%	4	8.7%	41	<0.001	45	97.8%	1	2.2%	0	0.0%	12	<0.001
2019	45	97.8%	1	2.2%	0	0.0%	9	<0.001	2	4.3%	37	80.4%	7	15.2%	15	<0.001	45	97.8%	1	2.2%	0	0.0%	9	<0.001
2020	39	84.8%	6	13.0%	1	2.2%	46	<0.001	1	2.2%	39	84.8%	6	13.0%	10	<0.001	40	87.0%	6	13.0%	0	0.0%	51	<0.001
2021	40	87.0%	6	13.0%	0	0.0%	43	<0.001	0	0.0%	41	89.1%	5	10.9%	0	<0.001	40	87.0%	6	13.0%	0	0.0%	41	<0.001
2022	43	93.5%	3	6.5%	0	0.0%	17	<0.001	0	0.0%	41	89.1%	5	10.9%	0	<0.001	36	94.7%	2	5.3%	0	0.0%	7	<0.001
2023	42	91.3%	4	8.7%	0	0.0%	22	<0.001	0	0.0%	41	89.1%	5	10.9%	0	<0.001	42	91.3%	4	8.7%	0	0.0%	22	<0.001

The Mann-Whitney U test was conducted to assess whether the overall distributions of age-standardized infectious disease burden rates differed significantly between males and females in Afghanistan aggregated across all 46 diseases and all 25 years (1999-2023). This test is appropriate for non-normally distributed rate data and does not require equal sample sizes, making it suitable for the YLL comparison, where minor discrepancies in sample sizes between sexes exist. The pooled Mann-Whitney U analyses were descriptive comparisons of the overall rate distributions between sexes and were not used as the primary longitudinal analysis because observations were repeated within etiology-by-sex series.

The results did not indicate a statistically significant overall difference between men and women (U=683751.5, Z=1.413, p=0.1577; N males=1150, N females=1150) for DALYs. The annual Wilcoxon analysis showed that men experienced significantly higher DALYs in multiple years, particularly in 2017 and 2021. These disparities were not large enough to yield a statistically significant aggregate difference when averaged across all 25 years between males and females, indicating annual tracking is essential to expose time-dependent disparities in burden.

However, for YLDs, women had a higher mean rank than men (Mean rank women=1183.48, Mean rank men=1117.52; U=623328.5, Z=-2.381, p=0.0172), a statistically significant difference. The findings are aligned with the pattern observed in the annual Wilcoxon analysis. This difference can be due to underlying unequal access to healthcare services.

For YLLs, the dataset entails 1,142 female and 1,150 male observations. The Mann-Whitney test does not require equal group sizes; therefore, it remains a valid test for comparing YLLs. The shortfall in female observations reflects eight missing enteric pathogen data points for females in 2022 in the GBD data source. Overall, results showed no statistically significant gender differences (U=678838.5, Z=1.401, p=0.1613); annual Wilcoxon analyses indicated that men experienced significantly higher YLLs during specific, conflict-affected years, such as 2017 in Afghanistan. The pooled Mann-Whitney findings are descriptive and differ from the GEE estimates because they do not adjust for etiology, calendar year, or correlation among repeated observations within etiology-by-sex series (see Table 2).

**Table 2:** Overall Sex-Based Differences in Age-Standardized Infectious Disease Burden in Afghanistan, 1999–2023 DALYs=Disability-adjusted life years; YLDs=years lived with disability; YLLs=years of life lost. The number of observations and percentage of total observations are reported for each sex. For DALYs and YLDs, males contributed 1150 observations (50.0%), and females contributed 1150 observations (50.0%), for a total of 2300 observations. For YLLs, males contributed 1150 observations (50.2%), and females contributed 1142 observations (49.8%), for a total of 2292 observations. The lower number of female YLL observations was due to eight missing enteric pathogen values in 2022 in the GBD 2023 source data. Mean rank=average rank of observations within each sex across the combined ranked distribution; sum of ranks=cumulative rank total for each sex; Mann–Whitney U=Mann–Whitney test statistic; Wilcoxon W=rank-sum statistic; Z=standardized normal approximation of the Mann–Whitney U statistic; p=asymptotic two-tailed p-value. A higher mean rank indicates that the group tended to have higher burden rates. Statistical significance was defined as p<0.05; p-values <0.001 are reported as p<0.001 [13].

Outcome	Sex	Number of observations	Percentage of total observations	Mean Rank	Sum of Ranks	Mann-Whitney U	Wilcoxon W	Z	p-value (2-tailed)
DALYs	Males	1150	50.0%	1170.07	1345576.5	683751.5	1300573.5	1.413	0.1577
DALYs	Females	1150	50.0%	1130.93	1300573.5	683751.5	1300573.5	1.413	0.1577
YLDs	Males	1150	50.0%	1117.52	1285153.5	623328.5	1285153.5	-2.381	0.0172
YLDs	Females	1150	50.0%	1183.48	1360996.5	623328.5	1285153.5	-2.381	0.0172
YLLs	Males	1150	50.2%	1165.79	1340663.5	678838.5	1287114.5	1.401	0.1613
YLLs	Females	1142	49.8%	1127.07	1287114.5	678838.5	1287114.5	1.401	0.1613

Generalized estimating equation models with an AR(1) working correlation structure were used to account for repeated annual observations within each etiology-by-sex series. DALY rates decreased by 5.354 per 100,000 population per year (95% CI: −8.178 to −2.530; p<0.001), YLL rates decreased by 5.301 per 100,000 population per year (95% CI: −8.105 to −2.498; p<0.001), and YLD rates decreased by 0.043 per 100,000 population per year (95% CI: −0.066 to −0.020; p<0.001). Sex-by-year interactions were not statistically significant for DALYs (B=−0.161; 95% CI: −4.208 to 3.886; p=0.938), YLLs (B=−0.165; 95% CI: −4.185 to 3.855; p=0.936), or YLDs (B=0.004; 95% CI: −0.027 to 0.035; p=0.792), providing no evidence that linear temporal trends differed by sex (see Table 3).

**Table 3:** Generalized Estimating Equation Analysis of Temporal Trends in Age-Standardized Infectious Disease Burden in Afghanistan, 1999–2023 DALYs=Disability-adjusted life years; YLDs=years lived with disability; YLLs=years of life lost. Separate GEE models were fitted for each age-standardized rate outcome (per 100,000 population). Each etiology-by-sex series was treated as a repeated subject, with calendar year as the within-subject variable. Models included etiology, sex, calendar year, and a sex-by-year interaction. An autoregressive first-order AR(1) working correlation structure was selected, and robust standard errors were used. The Year coefficient shows the estimated annual change in the rate for the reference sex category (females); a negative coefficient indicates a decline over time. The male=1×Year coefficient is the difference in the annual trend between male=1 and female=2; a p-value ≥0.05 indicates no evidence that sex-specific trends differed linearly over time. *Streptococcus pneumoniae* was the reference etiology, and female=2 was the reference sex category. The full etiology coefficient output is not shown because the table focuses on temporal trends and sex-by-year interactions. The DALY and YLD models each included 2,300 etiology-year-sex observations; the YLL model included 2,292 observations because female values for eight enteric etiologies were missing in 2022 in the GBD 2023 source data. Statistical significance was defined as p<0.05 [13].

Outcome	Parameter	B	Standard Error	95% Wald CI, Lower	95% Wald CI, Upper	Wald Chi-Square	df	p-value
DALYs	Year	-5.354	1.4408	-8.178	-2.530	13.809	1.000	<0.001
DALYs	Male=1×Year	-0.161	2.0650	-4.208	3.886	0.006	1.000	0.938
YLDs	Year	-0.043	0.0118	-0.066	-0.020	13.366	1.000	<0.001
YLDs	Male=1×Year	0.004	0.0159	-0.027	0.035	0.069	1.000	0.792
YLLs	Year	-5.301	1.4303	-8.105	-2.498	13.736	1.000	<0.001
YLLs	Male = 1×Year	-0.165	2.0510	-4.185	3.855	0.007	1.000	0.936

**Figure 1:**
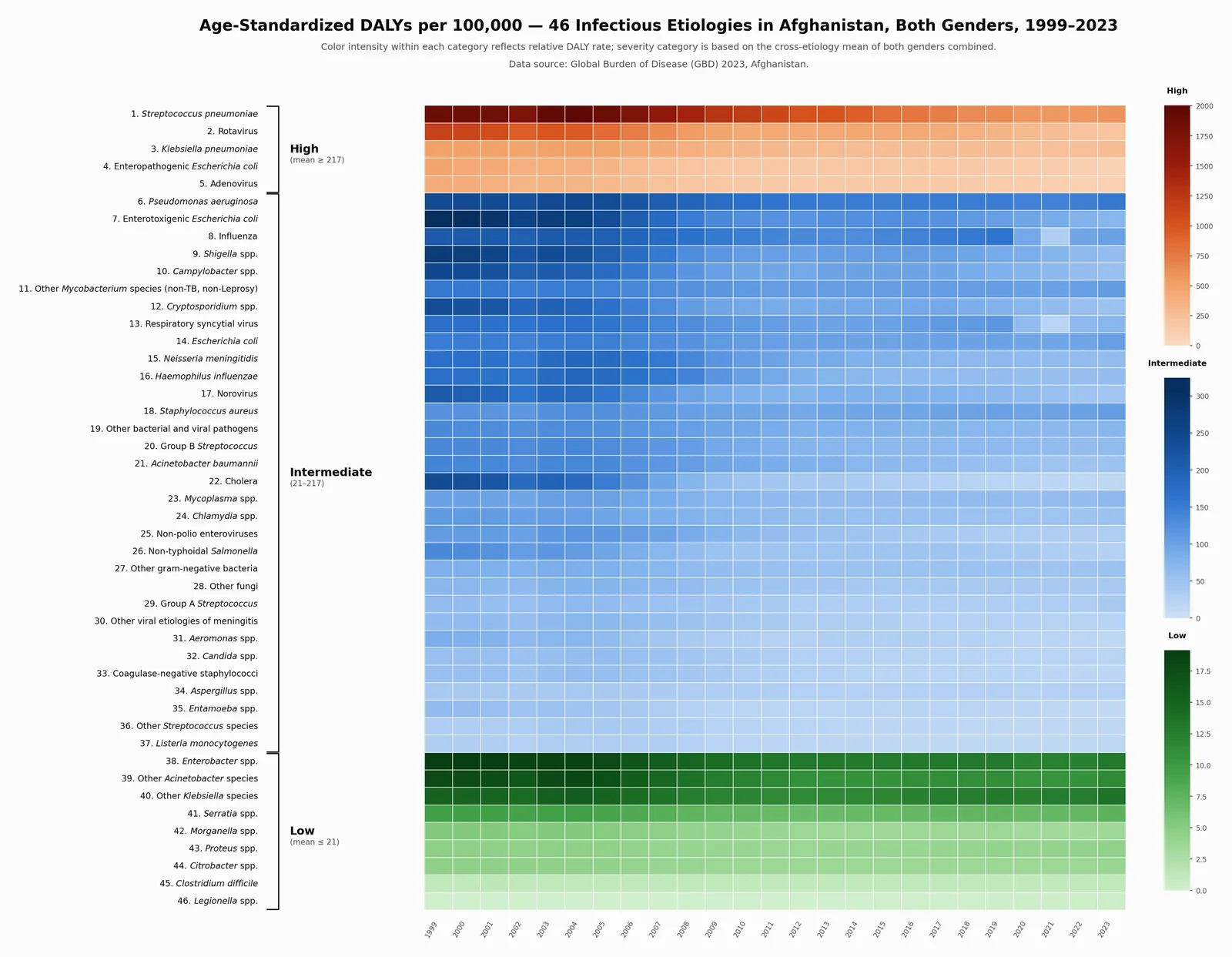
Age-standardized DALYs per 100,000 for 46 infectious etiologies in Afghanistan, both genders, 1999–2023, classified by burden severity Each row of the heatmap represents one of the 46 infectious etiologies from the GBD 2023 cause list, and each column represents a calendar year from 1999 to 2023. Cell color indicates the mean age-standardized DALY rate per 100,000 population for both genders combined. Color intensity within each severity category is scaled relative to the minimum and maximum mean DALY rates for etiologies within that category. Etiologies were grouped into three severity categories according to the cross-etiology mean DALY rate across all years: high-burden, shown with a red gradient, with mean DALY rates of 217 or more per 100,000 population and including five etiologies (10.9%); intermediate-burden, shown with a blue gradient, with mean DALY rates greater than 21 and less than 217 per 100,000 population and including 32 etiologies (69.6%); and low-burden, shown with a green gradient, with mean DALY rates of 21 or fewer per 100,000 population and including nine etiologies (19.6%). Severity thresholds were defined using ±0.5 standard deviations of the cross-etiology mean DALY rate (overall mean=119 per 100,000 population; standard deviation=196). Within each severity category, darker shades indicate a higher DALY burden relative to other etiologies in the same category. The three separate color bars on the right show the absolute DALY rate per 100,000 population for each severity category [13].

The heatmap provides a comprehensive overview of the age-standardized DALYs burden for 46 infectious etiologies in Afghanistan from 1999 to 2023 (Figure 1) for both genders. Based on the cross-etiology mean DALYs rate (119 per 100,000; SD=196), etiologies were classified into three severity bands: five high, 32 intermediate, and nine low burden etiologies.

The high-burden etiologies (red band), including *S. pneumoniae*, had the highest age-standardized DALY rate throughout the study period, followed by Rotavirus, *K. pneumoniae*, Enteropathogenic *E. coli*, and Adenovirus. These five etiologies displayed deep red coloring and sustained a high burden throughout the study period (1999-2023). A general lightening of color intensity is visible across the red band over time, indicating a gradual decline in DALYs among the highest-burden etiologies.

The intermediate-burden etiologies (blue band, n=32) showed greater heterogeneity in temporal patterns, such as *P. aeruginosa*, Enterotoxigenic *E. coli*, Influenza, and Shigella, which showed relatively darker blue cells in earlier years that faded progressively toward 2023, reflecting declining DALYs over time. Between 1999 and 2005, Cholera showed a pattern of darker cells, followed by a marked decrease.

The low-burden etiologies (green band, n=9) including *Enterobacter* spp., other *Klebsiella* species, other *Acinetobacter* species, *Serratia* spp., *Morganella* spp., *Proteus* spp., *Citrobacter* spp., *C. difficile*, and *Legionella* spp. maintained consistently low DALYs rates throughout the study period, as reflected by the uniformly pale green coloring across all years. The relative stability of these etiologies over 25 years suggests that while they account for a part of the burden landscape, they have not experienced major epidemic surges or systematic increases in Afghanistan during this period.

Overall, the heatmap shows that respiratory and enteric infections account for the majority of the most severe health burdens, while opportunistic and healthcare-associated pathogens make up the majority of the low-burden group. The gradual fading of color intensity from 1999 to 2023 across most severity bands is consistent with the significant decline in DALY rates estimated in the GEE model (Figure 1).

## Discussion

Annual paired comparisons indicated that male DALY and YLL rates were higher than female rates for many etiologies in numerous calendar years, whereas female YLD rates were higher for many etiologies. However, after adjustment for etiology and correlation among repeated annual observations, GEE models found no evidence that male and female burden rates changed at different linear rates between 1999 and 2023. This finding is consistent with broader trends observed in tuberculosis research in low- and middle-income countries, where men face nearly double the disease rates of women, largely because they are less likely to seek early medical attention or access diagnostic testing [14,15].

The apparent male excess in several annual comparisons after 2016 coincided temporally with periods of increased conflict in Afghanistan. This ecological temporal pattern may warrant further investigation. However, it cannot establish causation, and the GEE models did not identify a statistically significant linear sex-by-year interaction. Conflict-related disruptions to health services may have affected the prevention, diagnosis, and management of infectious diseases and may partly explain the observed increase in male burden [16]. Annual paired comparisons showed that female YLD rates exceeded male rates for many etiologies in each study year. This pattern is consistent with the possibility that sex-related social and healthcare factors may influence non-fatal infectious disease burden; however, the adjusted GEE model found no evidence that YLD trends differed linearly by sex over time [17]. Afghanistan specifically has been flagged as a country where cultural norms restrict women's autonomy to seek care [17-19]. Recent global reviews on gender and antimicrobial resistance similarly argue that gender-blind health policies fail to capture how social determinants, not just biology, drive variation in infectious disease outcomes between females and males [20,21]. This underscores why a sex-stratified, rather than pooled, approach was necessary to assess the burden of infectious diseases in Afghanistan.

The heatmap findings show *S. pneumoniae*, Rotavirus, and *K. pneumoniae* as the top high-burden etiologies. These findings are broadly consistent with the 2024 Lancet Infectious Diseases systematic analysis of 85 pathogens globally, which also identified respiratory and enteric pathogens, along with Gram-negative bacteria like *K. pneumoniae*, as major contributors to the global DALY burden, particularly in lower-income countries such as Afghanistan [22-24]. Taken together, these consistent patterns indicate the need to prioritize vaccine-preventable respiratory and enteric infections, as well as drug-resistant Gram-negative pathogens, in Afghanistan’s pathogen-specific infectious disease control initiatives. The modest yet statistically significant reductions in DALYs and YLLs observed broadly mirror the reported global trends [25,26]. This indicates substantial reductions in age-standardized DALYs for many communicable diseases. The observed declines may be consistent with improvements in prevention, vaccination coverage, and access to primary health care [27]. However, this study was not designed to identify the causal drivers of changes in infectious disease burden.

Ongoing systemic disparities in healthcare access in Afghanistan, where a substantial population remains underserved by primary healthcare, disproportionately affecting women of childbearing age, may provide a realistic contextual background for why progress has been inconsistent across the 46 studied etiologies [28]. These deficiencies may limit Afghanistan’s ability to benefit fully from global progress in infectious disease control and may contribute to unequal reductions in disease burden across population groups. Overall, the results of this study support the idea proposed in prior Afghanistan-specific GBD literature that continued investment in immunization, maternal-child health, and primary care access, layered with attention to conflict-driven disparities, will be essential to sustaining the gains reflected in the study's demonstrated temporal trends.

Strengths and limitations

This study offers a 25-year analysis of sex-specific infectious disease burden across 46 etiologies in Afghanistan, a setting with limited published evidence on long-term infectious disease burden. The analysis identifies etiologies, years, and sexes with the greatest burden and provides evidence to support resource prioritization by Afghan health authorities and international partners.

This study relied on secondary, aggregated GBD 2023 modeled estimates rather than raw surveillance or clinical data, so any biases embedded in the underlying GBD modeling framework carry over into our analysis. Female YLL data were missing for eight enteric etiologies in the 2022 dataset, resulting in an unequal sample size between women and men. Although the Mann-Whitney and Wilcoxon tests used are fairly robust to this degree of imbalance, the missing data could still introduce minor discrepancies into the assessment of that year. GEE models in this study used aggregated modeled GBD point estimates, and the analysis did not include the GBD 95% uncertainty intervals. The findings represent only population-average associations. Moreover, explanations related to conflict and barriers to healthcare access should be interpreted within the broader contextual frameworks, rather than as established causal mechanisms. The severity thresholds used to generate the heatmap (±0.5 SD of the cross-etiology mean DALY rate) were not for inferential purposes. The high, intermediate, and low burdens in the heatmap should be viewed only as a descriptive visualization tool. In lower-income settings such as Afghanistan, complete vital-registration data are often limited. GBD estimates rely substantially on statistical modeling, which may add uncertainty to this study’s findings.

## Conclusions

This study showed that Afghanistan's age-standardized infectious disease burden declined significantly in DALYs, YLDs, and YLLs over the 25-year study period after accounting for repeated annual observations within etiology-by-sex series. A small number of etiologies, particularly *S. pneumoniae*, rotavirus, and *K. pneumoniae*, accounted for a disproportionate share of the disease burden. These findings support the need to prioritize targeted vaccination, prevention, diagnostic, and treatment strategies. Annual paired comparisons showed higher male DALY and YLL rates across many etiologies in most calendar years, while female YLD rates were higher across etiologies in every study year. However, GEE analyses found no evidence that male and female burden rates followed different linear trends over the study period. The results support etiology-focused surveillance that is responsive to sex-specific patterns. However, differences observed between females and males should be interpreted cautiously and not assumed to represent different adjusted trends over time.
